# Paired Ear Creases of the Helix (PECH): A Possible Physical Sign

**DOI:** 10.7759/cureus.1884

**Published:** 2017-11-27

**Authors:** Pirunthan Pathmarajah, Christopher Rowland Payne

**Affiliations:** 1 General Medicine, Whipps Cross Hospital; 2 Dermatology, The London Clinic

**Keywords:** diagonal ear lobe crease, paired ear creases of the helix, frank's sign, coronary artery disease, metabolic syndrome

## Abstract

Diagonal ear lobe creases, often known as Frank’s sign, are a folding in the skin of the ear lobe. Many studies have found an association between diagonal ear lobe creases and coronary artery disease. To our knowledge, this is the first report of paired ear creases of the helix. They may have similar relevance to cardiovascular disease as the diagonal ear lobe creases. We report the case of a 68-year-old South Asian man with coronary artery disease and a diagonal ear lobe crease. On closer inspection of the auricle, he also had ear creases on the helix on the same side. We postulate that diagonal ear lobe creases and paired ear creases of the helix are formed due to pressure during sleep on a hard surface. The pathophysiological association of these creases to coronary artery disease and metabolic syndrome are not well understood. We report a new possible sign: paired ear creases of the helix which may have similar clinical significance as the diagonal ear lobe crease with respect to cardiovascular disease.

## Introduction

Diagonal ear lobe creases (DELC) are a folding in the ear lobe that is commonly referred to as Frank’s sign [1]. Not only does this have cosmetic implications, but it has been suggested that diagonal ear lobe creases are a recognised sign of susceptibility to coronary artery disease [2]. Reported here for the first time is a new possible physical sign: paired ear creases of the helix (PECH). They may have similar clinical relevance as DELC.

## Case presentation

**Figure 1:**
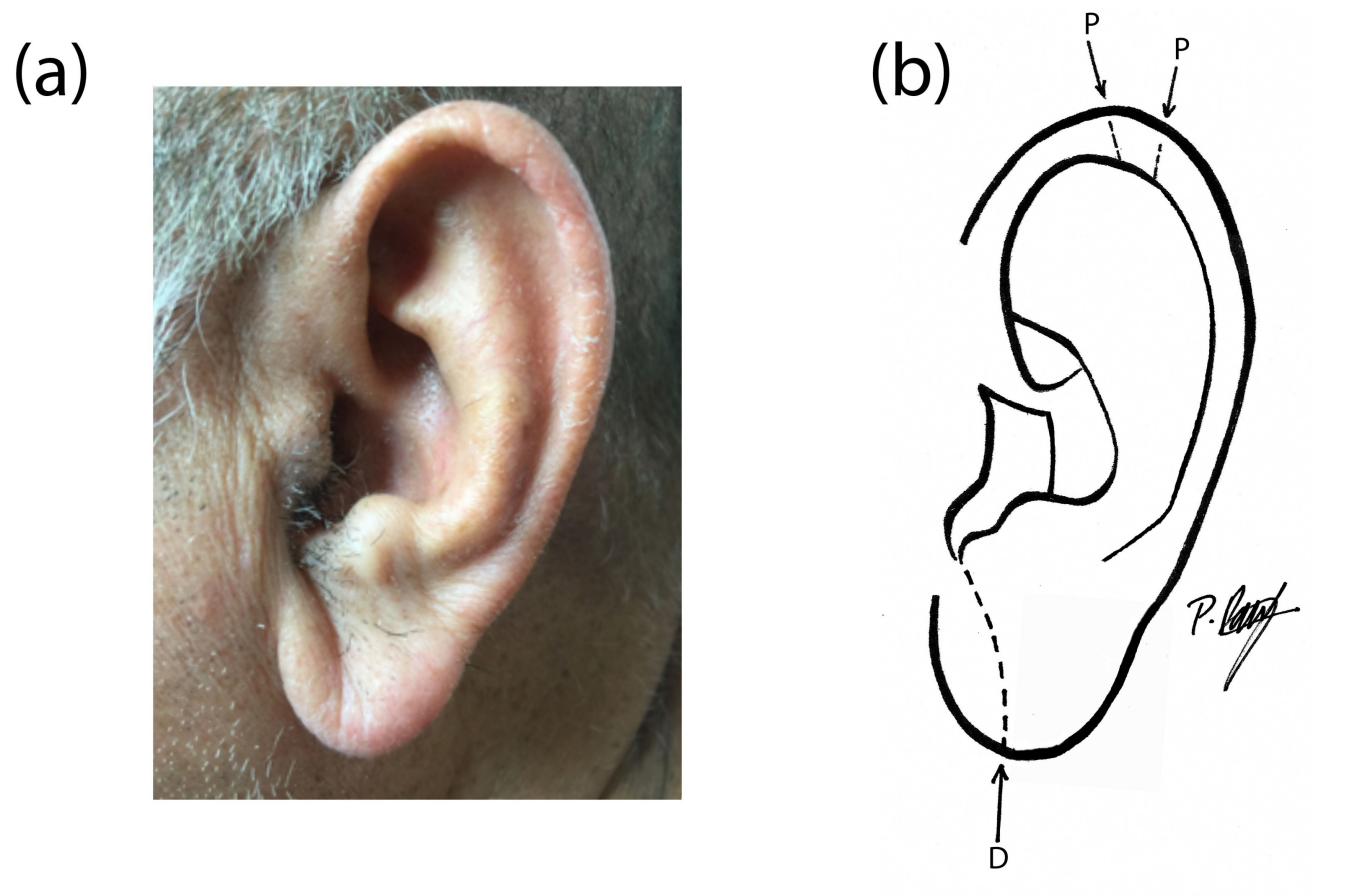
Photograph and diagram of the left ear (a) Diagonal earlobe crease and paired ear creases of the helix (photograph); (b) Diagonal earlobe crease (D) and paired ear creases of the helix (P) (diagram of photograph)

A 68-year-old South Asian man was referred for seborrhoeic dermatitis. He had a history of chronic lymphatic leukaemia, pityriasis capitis, and atopic xeroderma. On examination, he was noted to have end-stage androgenetic alopecia (AGA) (modified Norwood-Hamilton stage VII), a corneal arcus, and a metabolic syndrome characterized by obesity, hypertension, and hyperlipidaemia and complicated by coronary artery disease with supraventricular tachycardia. On the left helix, postero-superiorly, were paired ear creases, situated halfway between the upper pole of the helix and the auricular tubercle, the more inferior of the two being the deeper (Figure 1).

An ipsilateral DELC traversed from the tragus to the ear lobe margin. Inspection of the contralateral ear revealed no noteworthy creases.

## Discussion

Reports of DELC in the medical literature is becoming more noticeable. We postulate that DELC and PECH are the result of the ear being caught in between the rock of the cranium and the hard place of the mattress/pillow/hand. An alternative hypothesis is that the diagonal ear lobe creases may be the consequence of fat cheeks inducing a gravity-mediated valgus deformity of the hang of the ear lobe to such a degree that creases supervene. Many researchers have explored the association of this physical phenomenon with pathophysiological conditions. The pathophysiology between DELC and coronary artery disease still remains obscure. Biopsy results suggested that the ear lobe creases are a reflection of the extent of elastin loss and hence a reflection of the caliber of the coronary arteries [3]. Furthermore, Kang, et al. found in a cross-sectional study that ear lobe creases were correlated with metabolic syndrome after adjusting for cardiovascular risk factors [4]. PECH may have a similar association with coronary artery disease and metabolic syndrome which needs to be explored in further studies.

## Conclusions

In conclusion, there is rich literature concerning DELC. However, PECH are reported here for the first time. DELC is associated with coronary artery disease and the metabolic syndrome. The possibility exists that PECH, like DELC, may become a treasured tool to predict patients who are at risk of coronary artery disease and metabolic syndrome and allows clinicians to discuss the cardiovascular risk profile of asymptomatic patients. This warrants further investigation.
